# Fibromuscular dysplasia presenting with bronchial artery involvement

**DOI:** 10.1016/j.jvscit.2025.102108

**Published:** 2025-12-19

**Authors:** Ibrahim Miyanoorwala, Rashid Skeik, Jesse Manunga, Nedaa Skeik

**Affiliations:** a Minneapolis Heart Institute Research Foundation, Minneapolis, MN; b University of Minnesota, College of Liberal Art, Minneapolis, MN; c Minneapolis Heart Institute, Vascular Department, Allina Health, Minneapolis, MN

**Keywords:** Fibromuscular dysplasia, Bronchial artery aneurysm, Internal carotid, Celiac artery dissections

## Abstract

Fibromuscular dysplasia (FMD) is a rare noninflammatory, nonatherosclerotic vascular disorder associated with arterial beading, aneurysms, dissections, and rarely rupture, most commonly affecting the renal and carotid arteries in middle-aged women. We report a 75-year-old man with hypertension and hyperlipidemia found on computed tomography angiography to have celiac artery dissection, bilateral renal artery irregularities, and right bronchial artery aneurysms with a beading appearance consistent with FMD. The patient was managed conservatively with blood pressure control, antithrombotic therapy, and imaging surveillance. This case highlights the extremely rare involvement of bronchial artery in patients with FMD.

Fibromuscular dysplasia (FMD) is a nonatherosclerotic and noninflammatory arterial disease characterized by abnormal arterial wall development primarily affecting females.^1^ It most commonly affects the carotid and renal arteries, resulting in aneurysms, dissections, stenosis, and, in rare cases, rupture. FMD has been reported to involve multiple vascular beds, including the mesenteric, iliac, and coronary arteries.^2^^,^^3^ However, there have been very few reported cases of pulmonary vasculature involvement, particularly one other case involving the bronchial artery.^3^ It has been suggested that all patients with FMD should have screening images of neck, brain, chest, abdomen, and pelvis to ensure all arterial abnormalities are captured including possible intracranial aneurysms.^3^ We contribute to the limited body of knowledge on this rare presentation by sharing a case of an elderly man with FMD and right bronchial artery aneurysm. The patient agreed to having this case and associated images published.

## Case report

**Fig 1:**
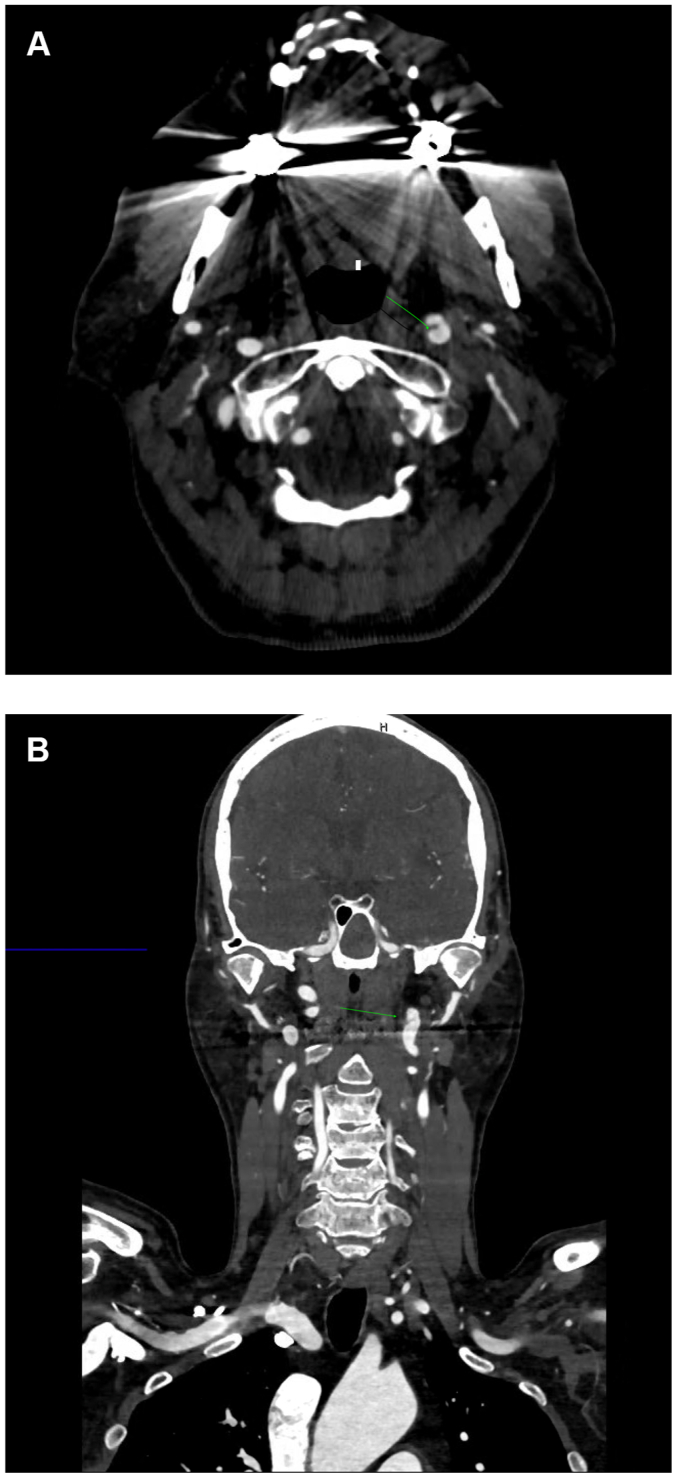
Computed tomography angiogram (CTA) transverse **(A)** and coronal **(B)** views showing left internal artery dissection, *green arrows*.

A 76-year-old man with a history of prediabetes, hyperlipidemia, and hypertension presented to our vascular clinic one month after a hospital admission for left middle cerebral artery stroke with concurrent left internal carotid artery (ICA) dissection that was managed medically. Vital signs revealed blood pressure of 136/74 mm Hg, and regular pulse of 64 beat per minute. Physical examination revealed normal cardiovascular examination with right hemiparesis. A complete blood count and a comprehensive metabolic panel were unremarkable. Lipid profile revealed a low-density lipoprotein level of 73 mg/dL. A repeat computed tomography angiogram (CTA) of neck and brain revealed stable left ICA dissection with bilateral ICA tortuosity consistent with FMD. There was no carotid arterial thrombosis or any significant concurrent stenosis (Fig 1, *A* and *B*).

**Fig 2:**
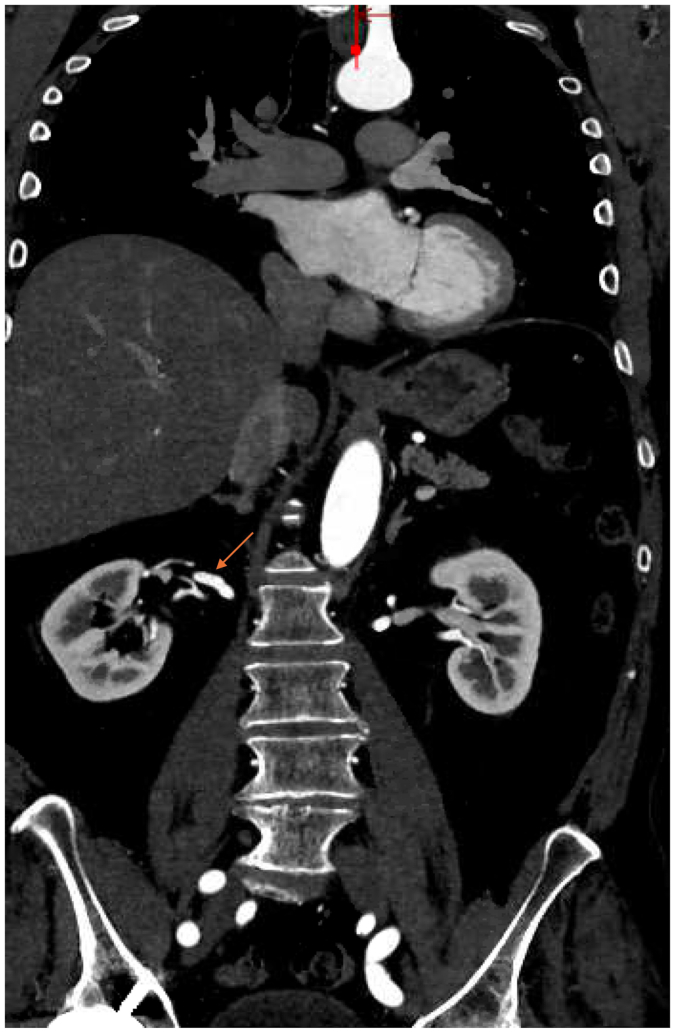
Computed tomography angiogram (CTA) sagittal view showing right renal artery beading appearance, *orange arrow*.

**Fig 3:**
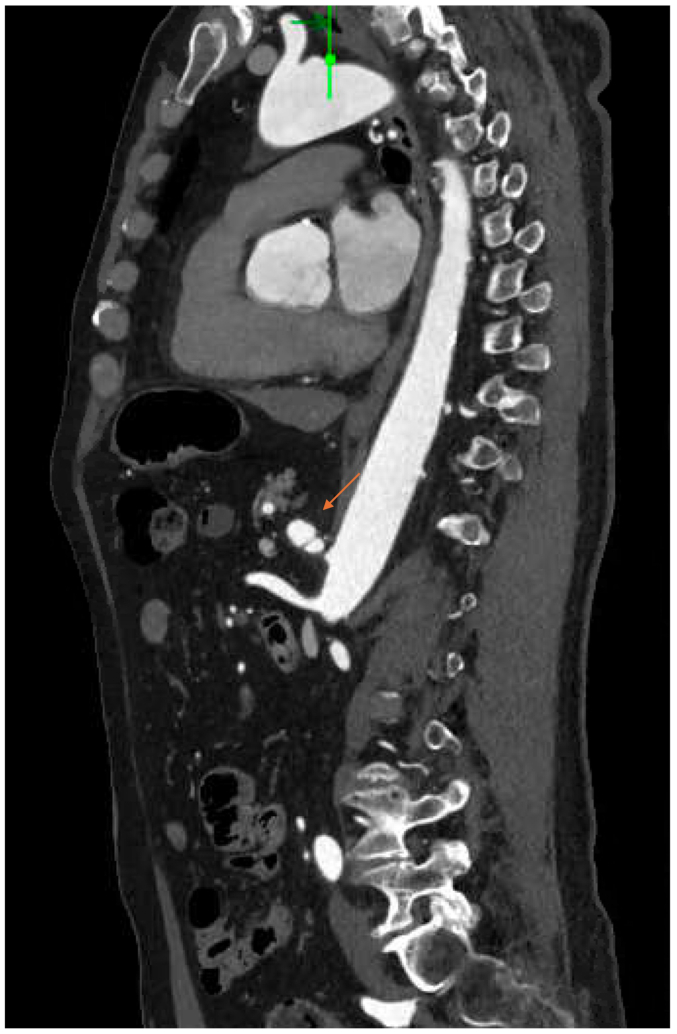
Computed tomography angiogram (CTA) sagittal view showing celiac artery dissection, *orange arrow*.

**Fig 4:**
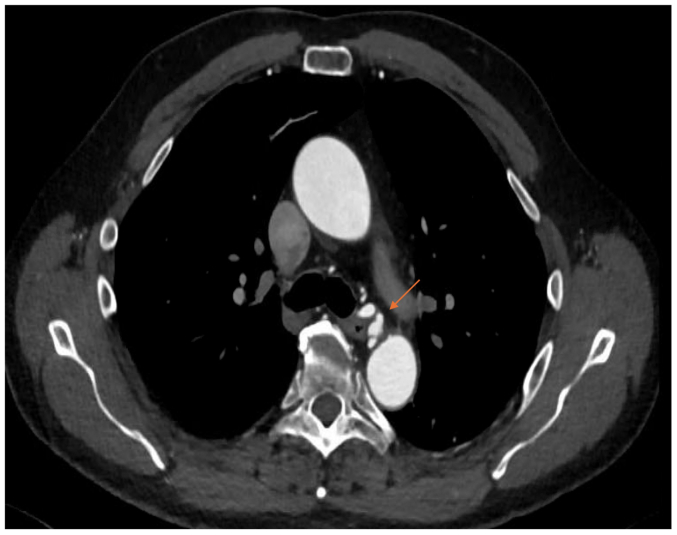
Computed tomography angiogram (CTA) transverse view showing right bronchial artery beading appearance, *orange arrow*.

**Fig 5:**
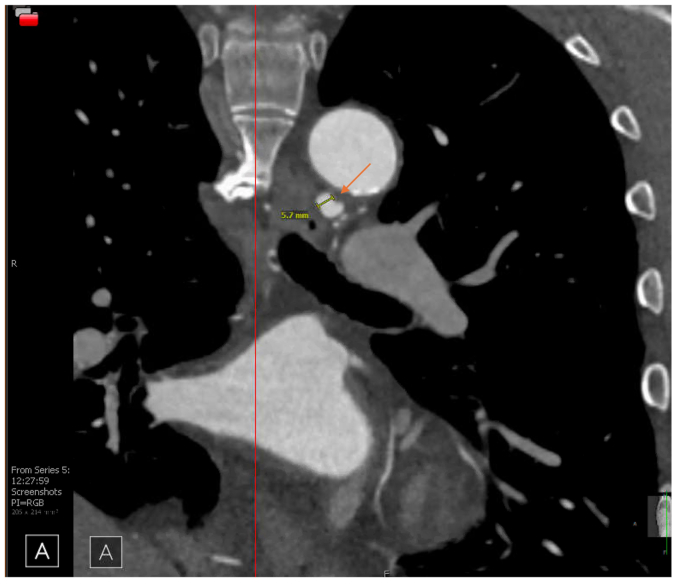
Computed tomography angiogram (CTA) coronal view showing right bronchial artery aneurysm measuring 5.7 mm, *orange arrow*.

**Fig 6:**
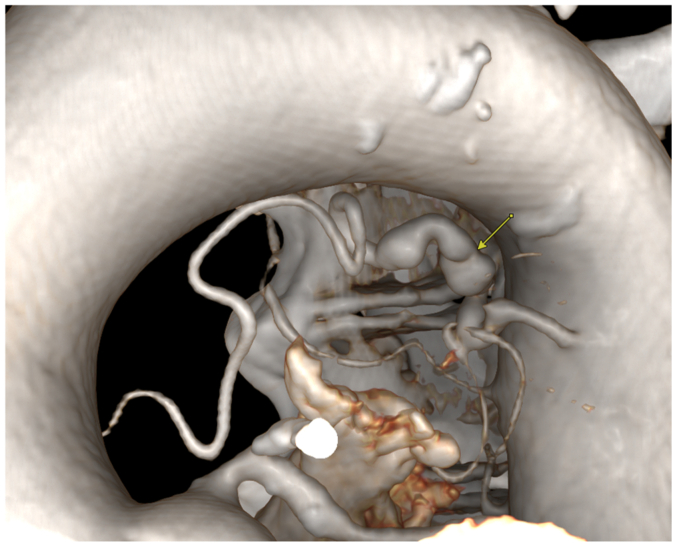
3 D reconstructed CTA images of the bronchial artery small aneurysms resembling string of beads, *yellow arrow*.

CTA of the abdomen and pelvis revealed bilateral renal artery beading, widely patent proximal celiac artery followed by distal dissection with concurrent pseudoaneurysm, measuring approximately 14 × 13 mm, and small aneurysms resembling beads involving the right bronchial artery (Fig 2, Fig 3, Fig 4, Fig 5, Fig 6). Although the left ICA dissection was thought to be the possible underlying cause for the patient's stroke because other etiologies such as atrial fibrillation or cardioembolic source were ruled out, an intervention was not offered because there was no flow-limiting lesion such as significant stenosis or concurrent thrombus. Because the other arterial findings were asymptomatic at that time, management included optimal blood pressure control with a goal of <120/80 mm Hg using lisinopril 10 mg daily, antiplatelet therapy with aspirin 81 mg/day, and statin with atorvastatin 40 mg every night. During the next 3 years, the patient was followed by a vascular medicine specialist and a vascular surgeon, and had repeated CTA at 6- and 12-month intervals. Future plan is to continue surveillance with magnetic resonance angiogram at 1- to 2-year intervals or sooner if he becomes symptomatic.

### Literature search

A literature review was conducted using PubMed, applying the keywords “fibromuscular dysplasia” and “pulmonary,” and “fibromuscular dysplasia” and “bronchial.” Filters were applied to limit results to cases reported in English.

## Discussion

FMD is a rare, nonatherosclerotic, noninflammatory vascular disorder that was first described by Leadbetter and Burkland in 1938.^1^ The true prevalence of FMD in the general population remains uncertain, because it is often underdiagnosed; however, a recent large-scale analysis in the United States estimated a prevalence of approximately 12 cases per 100,000 persons, with higher occurrence in women (80%-90%) and Caucasians.^2^^,^^3^

Historically, FMD was classified by histopathology and categorized into medial, intimal, and perimedial subtypes. The most common type of FMD is medial fibroplasia, which appears as a string of beads pattern, accounting for 80% to 90% of cases.^4^^,^^5^ In contrast, intimal fibroplasia accounts for approximately 10% of all FMD cases, produces a smooth, concentric narrowing of the vessel, and is most commonly observed in children.^6^ Perimedial fibroplasia is rare and is characterized by smaller and fewer beads compared with medial fibroplasia.^4^

However, the histopathological classification of FMD is no longer used in clinical practice, and current diagnostic criteria instead rely on angiographic appearance. Although catheter-based angiography is the gold standard for imaging the location and morphology of FMD, noninvasive imaging techniques such as CTA or magnetic resonance angiography are preferred for initial evaluation.^3^ Using these imaging modalities, FMD can be categorized as focal or multifocal.^3^ Focal FMD presents as a concentric or tubular stenosis and may occur in any part of the artery.^3^^,^^7^ In contrast, multifocal FMD presents with alternating areas of stenosis and dilation, producing the string of beads pattern, and usually occurs in the mid and distal segments of the artery.^3^

The etiology of FMD is not fully understood and is both sporadic and familial, with an autosomal-dominant inheritance pattern being suggested in some families.^8^^,^^9^ However, only a minority of patients in modern registries have an affected relative, reflecting incomplete penetrance and underdiagnosis of FMD.^10^^,^^11^ Genetic studies of genes associated with other arteriopathies have not identified a clear overlap with FMD, but a recent genome-wide association study revealed a single nucleotide polymorphism (rs9349379-A) in the *PHACTR1* locus, which confers an estimated 1.4-fold increased risk of developing FMD.^12^, ^13^, ^14^ This variant has also been associated with cervical artery dissection, hypertension, and migraine, which are commonly seen FMD-associated abnormalities.^3^ These findings suggest a complex genetic basis for FMD, and further studies are needed to identify which genes can be clinically useful to predict high risk.^3^

FMD most frequently affects the renal and extracranial carotid and vertebral arteries.^3^ Nonetheless, it can manifest in nearly all arterial beds, and multivessel disease is common, with more than one-half of patients in the US Registry demonstrating FMD in two or more vascular territories.^3^^,^^10^ Hypertension is the predominant clinical manifestation, reflecting approximately 75% of patients with renal artery involvement.^10^^,^^11^ Cerebrovascular disease is also frequent and can present with migraine, pulsatile tinnitus, transient ischemic attack, or stroke.^3^ Beyond these primary sites, FMD has been reported in less typical areas, such as the mesenteric, iliac, and coronary arteries.^15^, ^16^, ^17^ Involvement of the pulmonary vasculature is exceedingly rare, with very few reports describing bronchial artery FMD, highlighting the importance of our report in expanding the spectrum of FMD presentations.^18^

In 2020, Stejskal et al^18^ first documented the association between FMD and the bronchial artery, reporting the case of a 40-year-old woman with moyamoya disease and concurrent FMD of intrapulmonary bronchial arteries, identified incidentally on autopsy. Histological findings were consistent with the medial hyperplasia subtype of FMD, which is most commonly seen in the renal arteries of young women.^18^

A literature search using the terms “fibromuscular dysplasia” and “pulmonary” yielded 45 articles published between 1976 and March 2025, of which 9 cases met the criteria for confirmed or suspected pulmonary FMD. The remaining 36 articles were excluded because they either did not describe pulmonary arterial involvement or lacked sufficient histopathological or angiographic confirmation to establish pulmonary FMD. The search using the terms “fibromuscular dysplasia” and “bronchial” yielded only a single confirmed case report of a 40-year-old woman who passed away of moyamoya-related complications.^18^ In total, to the best of our knowledge, ten cases of pulmonary or bronchial artery FMD have been reported (Table). Most were managed conservatively with medical therapy, similar to our case. Multiple cases of pulmonary artery involvement had fatal pulmonary hemorrhage, creating a challenge for managing these rare cases. Notably, our case contributes to the limited literature describing bronchial artery involvement in FMD and provides a clinically confirmed diagnosis in a living male patient. Having diverse presentations and treatment options emphasizes the need for more research to standardize the diagnostic process and management strategies for these patients.

**Table:** Reported cases of fibromuscular dysplasia (*FMD*) and FMD-like lesions in the pulmonary and bronchial arteries

Patient No.	Age/sex	Investigator	Arterial FMD involvement	Presenting symptoms	Management	Outcome
1	52/Female	Sarbia et al.^19^ (1993)	Pulmonary arteries (angiodysplasia with features suggestive of FMD)	Fatigue, blood-streaked sputum, retrosternal burning, and interscapular pain	Right upper and middle lobectomy; diagnosis confirmed postmortem	Fatal massive hemoptysis
2	13/Male	Fukuhara et al.^20^ (1996)	Pulmonary arteries, renal arteries, and femoral arteries	Abnormal ECG and hypertension; pulmonary hypertension identified during evaluation	Percutaneous transluminal angioplasty, renal autotransplantation, emergency nephrectomy	Death suspected of a cerebrovascular lesion not confirmed by autopsy
3	7/Female	Fukuhara et al.^20^ (1996)	Pulmonary arteries, common iliac arteries, left ICA, right middle cerebral artery, and right basilar artery	Abnormal ECG, exertional syncope, headaches, and general malaise; pulmonary hypertension identified during evaluation	Warfarin, prostacyclin	Alive, stable, and occasional mild headaches
4	69/Male	Campman et al.^21^ (2000)	Pulmonary arteries	Pleuritic chest pain (initially back pain), syncope, fatigue, dyspnea, and productive cough	Supportive care for presumed pneumonia; no management of FMD, autopsy finding	Fatal hemothorax and lung hemorrhage
5	10/Male	de Vries et al.^22^ (2003)	Pulmonary arteries	Dizziness, palpitations, paleness, and exertional syncope; pulmonary hypertension identified during evaluation	Balloon dilations of the stenotic pulmonary arteries	Fatal pulmonary hemorrhage shortly after balloon dilations
6	15/Female	Ou et al.^23^ (2006)	Pulmonary and renal arteries	Evaluation for progressive pulmonary hypertension initially considered idiopathic; no pulmonary symptoms	Percutaneous balloon angioplasty and surgical revascularization for renal artery involvement; pulmonary hypertension treatment not reported	NR
7	20/Female	Yano et al.^24^ (2010)	Pulmonary arteries, left subclavian artery, left common carotid artery, celiac artery, superior mesenteric artery, and cerebral arteries	Worsening dyspnea and chest pain prompting reevaluation of pulmonary hypertension	Bosentan, warfarin, sildenafil	Symptoms worsened after 18-month follow-up
8	41/Male	Lam et al.^25^ (2020)	Pulmonary, iliac, celiac, renal, and coronary arteries	Right thigh swelling and pain leading to diagnosis of deep vein thrombosis; no pulmonary symptoms	Endovascular repair of iliac artery aneurysm, medical therapy, surveillance imaging	Remained asymptomatic at 6-year follow-up
9	40/Female	Stejskal et al.^18^ (2020)	Intrapulmonary bronchial arteries	Recurrent cerebral ischemia and progressive vision loss; no pulmonary symptoms	No management, incidental autopsy finding in Moyamoya disease	Death due to Moyamoya complications
10	17/Female	Chen et al.^26^ (2023)	Pulmonary, intracranial carotid, vertebral, left internal carotid, celiac, superior mesenteric, and renal arteries	Severe uncontrolled hypertension and hemoptysis	Percutaneous transluminal angioplasty for renal artery stenosis, medical therapy for pulmonary hypertension	Stable after 3 years, blood pressure was normal, and pulmonary artery hypertension was improved

*ECG,* Electrocardiogram; *ICA,* internal carotid artery; *NR,* not reported.

## Conclusions

Although FMD can affect many arterial beds, involvement of the bronchial artery is extremely rare. This case adds to the limited literature describing this unique arterial involvement. Atypical manifestations of FMD must be considered, especially in male patients, where the condition is less commonly observed. Currently, no guidelines exist for managing FMD in these unusual sites. Given the limited data, conservative management has been the most commonly reported treatment for these rare involvements. As we call for more research to address this unique FMD arterial involvement, we suggest individualized treatment based on risk and benefit.

## Funding

None.

## Disclosures

None.
